# Synthesis of complex rare earth nanostructures using *in situ* liquid cell transmission electron microscopy†

**DOI:** 10.1039/c9na00197b

**Published:** 2019-04-18

**Authors:** Caitlin A. Taylor, Tina M. Nenoff, Sarah H. Pratt, Khalid Hattar

**Affiliations:** Sandia National Laboratories P.O. Box 5800, MS-1056 Albuquerque New Mexico 87185 USA khattar@sandia.gov +1 505 845 9859; Explora 1701 Mountain Road NW Albuquerque New Mexico 87104 USA

## Abstract

Energy and cost efficient synthesis pathways are important for the production, processing, and recycling of rare earth metals necessary for a range of advanced energy and environmental applications. In this work, we present results of successful *in situ* liquid cell transmission electron microscopy production and imaging of rare earth element nanostructure synthesis, from aqueous salt solutions, *via* radiolysis due to exposure to a 200 keV electron beam. Nucleation, growth, and crystallization processes for nanostructures formed in yttrium(iii) nitrate hydrate (Y(NO_3_)_3_·4H_2_O), europium(iii) chloride hydrate (EuCl_3_·6H_2_O), and lanthanum(iii) chloride hydrate (LaCl_3_·7H_2_O) solutions are discussed. *In situ* electron diffraction analysis in a closed microfluidic configuration indicated that rare earth metal, salt, and metal oxide structures were synthesized. Real-time imaging of nanostructure formation was compared in closed cell and flow cell configurations. Notably, this work also includes the first known collection of automated crystal orientation mapping data through liquid using a microfluidic transmission electron microscope stage, which permits the deconvolution of amorphous and crystalline features (orientation and interfaces) inside the resulting nanostructures.

## Abstract 2

Energy and cost efficient synthesis pathways are important for the production, processing, and recycling of rare earth metals necessary for a range of advanced energy and environmental applications.

## Graphical abstract

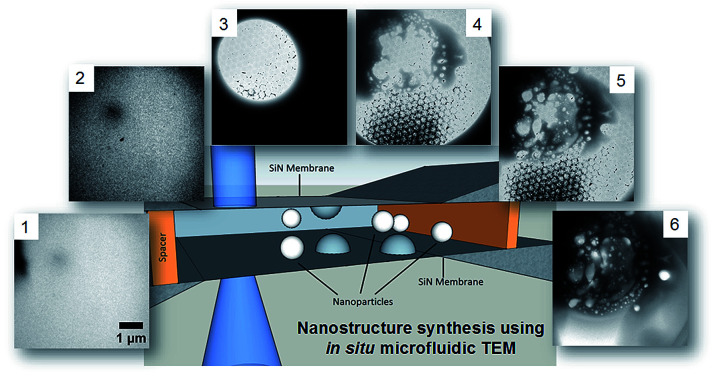

## Introduction

Rare earth compounds have become ubiquitous in a range of modern applications ranging from cellular communication to alternative energy sources.^1,2^ As a result of this increased demand and the globally distributed low concentration, there has been a heightened urgency to increase the efficiency and safety associated with mining, processing, utilizing and ultimately recycling these compounds.^3,4^ For many of these advanced applications, both the phase and morphology of the rare earth compound must be controlled down to the nanometer scale for proper operation. The development of energy and cost efficient (*e.g.* room temperature) aqueous processing requires a fundamental understanding the basic physics and chemistry governing the structural evolution that occurs during processing.^5^

In addition to direct rare earth applications, many lanthanide compounds are used as actinide surrogates. The study of actinides and their role in civilian nuclear energy is vital as they need to be isolated from nuclear waste streams and properly disposed to avoid environmental contamination^6,7^ Actinides can be found as ions dissolved in the aqueous waste streams. To safely retrieve for disposal or even possible reuse, various capture methods have been developed.^8^

Gamma irradiation is one of the few radiolysis methods explored for synthesizing lanthanide nanoparticles.^9–13^ Under gamma irradiation, hydrated electrons, hydrogen atoms and hydroxyl radicals are created from H_2_O in the aqueous reaction solution. The hydrated electrons then reduce the metal (M) ions in solution to produce metal nanoparticles (see eqn (1)):

1 M^*n*+^ + *n*e^−^_aq_ → M^0^

This reaction path has been shown to be applicable in reduction of uranyl (UO_2_^2+^) ions to UO_2_ nanoparticles *via* radiolysis through gamma irradiation^9–11^ In a complimentary nature, *in situ* liquid cell techniques have been shown to be a powerful tool to elucidate the chemistry and physics governing the size and morphology of noble metal nanoparticle formation.^14–17^

Herein, the rapidly maturing field of *in situ* microfluidic Transmission Electron Microscopy (TEM)^18–20^ was applied to rare earth chemistry in order to study rare earth nanostructure growth and crystallization. In these experiments, an electron beam was converged into a microfluidic TEM stage filled with rare earth salts dissolved in solution. Nanostructure formation due to electron beam irradiation induced radiolysis was observed *in situ* by repeatedly converging the electron beam inside the fluid. Use of the *in situ* microfluidic TEM stage allows for real-time observation of nanostructure nucleation pathways and the determination of crystallization rates, as a function of electron beam dose using electron diffraction.^21^ Several previous studies have investigated nanostructure growth and diffusion *in situ* using a microfluidic cell in a range of materials including, but not limited to: Au,^16^ Ag,^15^ PbS,^14^ Pd,^17^ Pb,^22^ and CaCO_3_.^23^ However, to the best of our knowledge, this is the first study to quantify the nanostructure formation and crystallization process of rare earths inside the TEM. Chemical reactions occurring under electron beam induced radiolysis are more complex and are detailed elsewhere.^17^ In the end, the same reduction mechanism of the salt to a pure metal structure is expected. The specific reaction in eqn (2) is predicted to occur after exposure of salt solutions to the electron beam, where Ln = Y, La, or Eu:

2 Ln^3+^ + 3e^−^_aq_ → Ln^0^(s)

Despite the simplistic prediction, a range of structures were synthesized, including pure metals, salts, and oxides due to the competing electron beam enhanced precipitation and oxidation mechanisms. Through the reaction in eqn (2), redox potentials for each rare earth are as follows: La = −2.379 eV, Eu = −1.991 eV, and Y = −2.38 eV.^24^ In addition to real-time characterization of nanostructure formation, this work presents the first study to utilize Precession Electron Diffraction (PED) in a fluid environment to produce Automated Crystal Orientation Maps (ACOM), which permitted the spatial deconvolution of the amorphous and crystalline components of as-synthesized nanostructures. ACOM maps provide a rapid and high spatial resolution method to deconvolute phase in a microfluidic environment compared to previous techniques including the recently developed *in situ* liquid cell electron diffraction tomography.^25^

### Experimental methods

The instrumentation used to explore the response of these rare earth salts included the *in situ* ion irradiation TEM (I^3^TEM), a 200 kV JEOL JEM 2100 TEM^26^ and a Poseidon *in situ* microfluidic TEM stage developed by Protochips, Inc.^27^ The microfluidic stage consists of two Si chips with 50 nm-thick SiN_*x*_ windows, each 400 × 50 μm in dimension. Two O-rings seal the liquid cell and a metal plate (with a small hole for the electron beam to enter), which is screwed on top. The cell has two inlets and one outlet running from the stage tip to the end of the holder. Experiments were performed in both “closed cell” and “flow cell” modes of operation.

Three rare earth salt solutions were explored with the *in situ* liquid cell TEM: yttrium(iii) nitrate (Y(NO_3_)_3_·4H_2_O), lanthanum(iii) chloride hydrate (LaCl_3_·7H_2_O), and europium(iii) chloride hydrate (EuCl_3_·6H_2_O). Concentrations of salt solutions were mixed prior to TEM and microfluidic studies using the same procedure for the closed cell and flow cell experiments. All reagents were purchased from Aldrich. For Y(NO_3_)_3_·4H_2_O, 1.009 g (346.98 g mol^−1^) of Y(NO_3_)_3_·4H_2_O was mixed in 10 mL H_2_O = 0.291 M (M; mol liter^−1^) solution. For LaCl_3_·7H_2_O, 0.959 g (371.37 g mol^−1^) of LaCl_3_·7H_2_O was mixed in 20 mL H_2_O = 0.129 M (more dilute to dissolve all the salt without heating). For EuCl_3_·6H_2_O, 0.509 g (366.41 g mol^−1^) of EuCl_3_·6H_2_O was mixed in 10 mL H_2_O = 0.139 M. Solutions were diluted with deionized (DI) water before being pipetted onto the TEM stage for the closed cell experiments. The amount of DI water used to successfully dilute the solutions used in the closed cell experiments varied depending on the time between solution preparation and the *in situ* TEM experiment. Multiple diluted solutions were prepared and iterated inside the TEM to determine the optimum concentration for *in situ* imaging of nanostructure growth and crystallization. If the prepared solution was too concentrated, the solution would immediately form large crystals several hundred microns in size upon exposure to the electron beam. In some cases, the prepared solution would result in a highly viscous and electron beam opaque liquid immediately after electron beam exposure. In other cases, several hundred nanometer thick crystals that were visible inside the TEM, but too thick to image, would immediately form. If nanostructures were not detected after irradiation under the electron beam, then the solution was assumed too strongly diluted, and subsequent higher concentration solutions were prepared. Several solutions of varying salt concentration were prepared until the optimum dilution was identified. In flow cell experiments, liquid was flowed through inlets on the back of the stage during electron beam exposure. First, the stage was assembled and deionized water was flowed to both confirm proper functionality and to align the TEM for imaging through liquid. Next, the appropriate salt solution was drawn into a 5 mL syringe and connected to the syringe pump and tubing. During the nanostructure growth stage of the experiment, the salt solution was flowed with a 100–300 μL h^−1^ rate, depending on solution.

In closed cell experiments, a drop of liquid was pipetted from a syringe directly onto the bottom microfluidic chip of the TEM stage. Liquid was not flowed through the stage during closed cell experiments, and the liquid remained nominally static. Any nanostructure motion observed was thus due to interaction with the electron beam or stage vibrations. Selected Area Diffraction (SAD) patterns were recorded *in situ* when possible, providing quantification of crystallization, as a function of electron beam dose. SAD patterns were composed of rings in all cases, indicating arrays of nanoparticles or nanocrystals formed. In some cases, crystallization occurred without any visible alterations to the nanostructure. *In situ* electron diffraction and ACOM phase identification was only used in a closed cell environment, where the static solution remained more stable during the nucleation stages compared to under liquid flow. In both closed cell and flow experiments, very little changes in microstructure were observed without converging the electron beam. The electron beam was consequently repeatedly converged, effectively pulsing a high intensity, non-periodic beam of electrons into the solution. The converged electron flux was measured directly from the TEM screen before each closed cell experiment. Total electron doses were calculated based on time under the converged electron beam.

Video was recorded at adequate magnification to observe features that appeared to undergo the most change under the electron beam. In some cases, higher magnification images were taken after the *in situ* experiments to identify smaller nanoparticles. Particle sizes were quantified using the Analyze Particles feature of ImageJ.^28^ Global phase identification was done by comparing radius ratios calculated from SAD ring patterns to the inverse *d*-spacing ratios taken from Powder Diffraction Files (PDF) of known structures. All electron diffraction patterns utilized for phase identification are provided in the supplemental file. The supplemental file also contains tables with the measured radius ratio, and the percent difference between the measured and database radius ratios, for each synthesized compound. The rare earth salt and pure rare earth metal crystal structures were considered most likely candidates and were compared first, followed by metal oxide crystal structures. PDFs were taken from the Inorganic Crystal Structure Database (ICSD)^29,30^ and crystal structure images were generated using CrystalMaker®.

The work presented here is the first known success at attempted ACOM through liquid in the microfluidic TEM cell. The NanoMEGAS PED system was used for this analysis. ACOM was performed post nanoparticle formation utilizing the lowest possible beam intensity in the TEM. This beam current condition, which could not be measured using the screen on the JEOL JEM 2100, has been previously used to characterize complex nanoparticles^31^ and ensures that the electron beam intensity during ACOM scans were significantly less than during the *in situ* experiments, minimizing the possibility of additional reduction reactions. Nanostructures were determined to be stable in terms of growth and crystallinity prior to ACOM analysis. Additionally, due to the novelty of the procedure, some of the challenges encountered in these experiments and future potential are discussed below.

## Results and discussion

Yttrium, lanthanum, and europium salt solutions were dissolved and irradiated with the 200 keV electron beam *in situ*, with the microfluidic stage in “closed” and “flow” cell configuration. Resulting microstructures and possible mechanisms are described below. Crystallinity was achieved for each salt solution, but crystalline components of the nanostructures were difficult to distinguish from amorphous fluid, microfluidic cell windows, and amorphous components of the nanostructures.

### Closed cell experiments

#### Y(NO_3_)_3_·4H_2_O

First, a 0.026 M diluted solution, prepared from an 8 d old Y(NO_3_)_3_·4H_2_O salt solution, was imaged using a beam flux of 1.6 × 10^−7^ e^−^ Å^−2^ s^−1^. Large, thick, amorphous particles formed under the electron beam. Imaging and diffraction were difficult because of the thickness. A fresh (0 d old) salt solution was prepared and diluted to the same concentration. The experiment was repeated, this time using a flux of 2.6 × 10^−7^ e^−^ Å^−2^ s^−1^, and the SiN_*x*_ windows burst during electron beam exposure. The cause of this may be due to higher electron beam flux causing larger salt crystals to form in the second experiment, or additional liquid being pipetted into the cell. Next, a 0.018 M diluted solution was prepared from the fresh (0 d old) salt solution. Using the same electron beam flux, the SiN_*x*_ windows become plated with amorphous material under the electron beam. A 0.014 M solution was also prepared from the fresh (0 d old) solution and irradiated with the same beam flux. In this case, thick, whip-like structures, which were possibly partially crystallized, formed inside the solution.

**Fig. 1:**
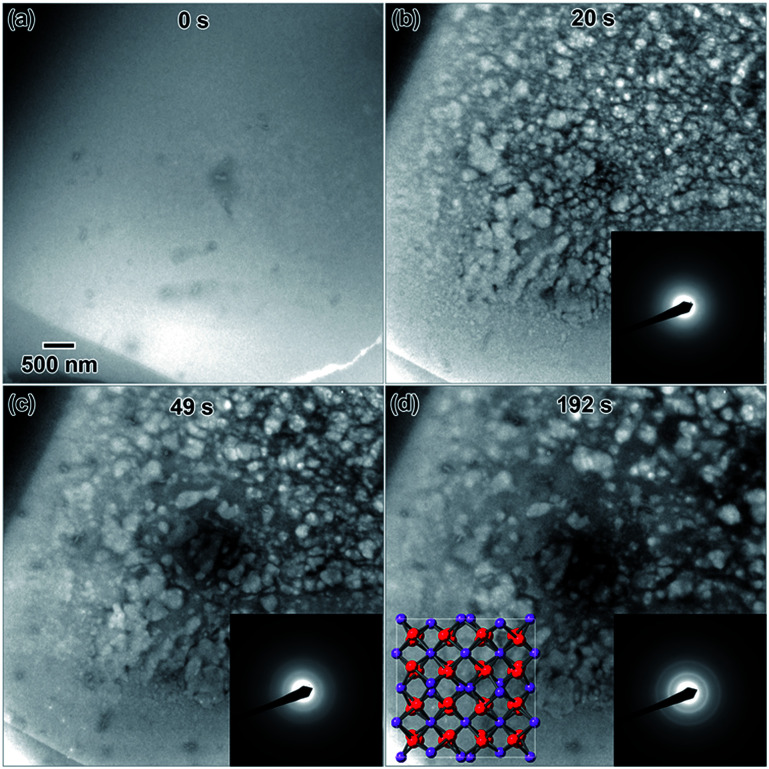
*In situ* images of nanostructure formation in 0.009 M Y(NO_3_)_3_·4H_2_O solution irradiated using a converged e^−^ beam flux of 2.6 × 10^−7^ e^−^ Å^−2^ s^−1^. Frames (a)–(d) show the progression of nanostructure formation from an empty solution as a function of the total time under the converged e^−^ beam. SAD patterns were taken *in situ* after the same total irradiation time. The final crystal structure was determined to be Y_2_O_3_ using the SAD in (d). A drawing of the Y_2_O_3_ crystal structure is shown in the [001] viewing direction as an inset in (d), where red = oxygen and purple = yttrium.

Highly crystalline nanostructures were finally achieved using a 0.009 M solution (Fig. 1), which was prepared using a 1 day old salt solution and irradiated to the same 2.6 × 10^−7^ e^−^ Å^−2^ s^−1^ flux. Fig. 1(a) shows the empty fluid prior to converging the electron beam. Note that with regular imaging (*i.e.* beam not converged), nanostructures do not form. Fig. 1(b) shows the globular structures that formed after 20 s under the converged electron beam. The diffraction pattern appeared amorphous. Most of the fluid in the region where nanostructures formed appeared to be solid after 20 s. After 49 s, some of the smaller particles appeared to coalesce to form larger structures, shown in Fig. 1(c). At this point, the electron diffraction pattern showed some signs of crystallinity. After 192 s, in Fig. 1(d), some additional coalescence of smaller particles into larger particles appeared to occur, especially near the center of nanostructure.

**Fig. 2:**
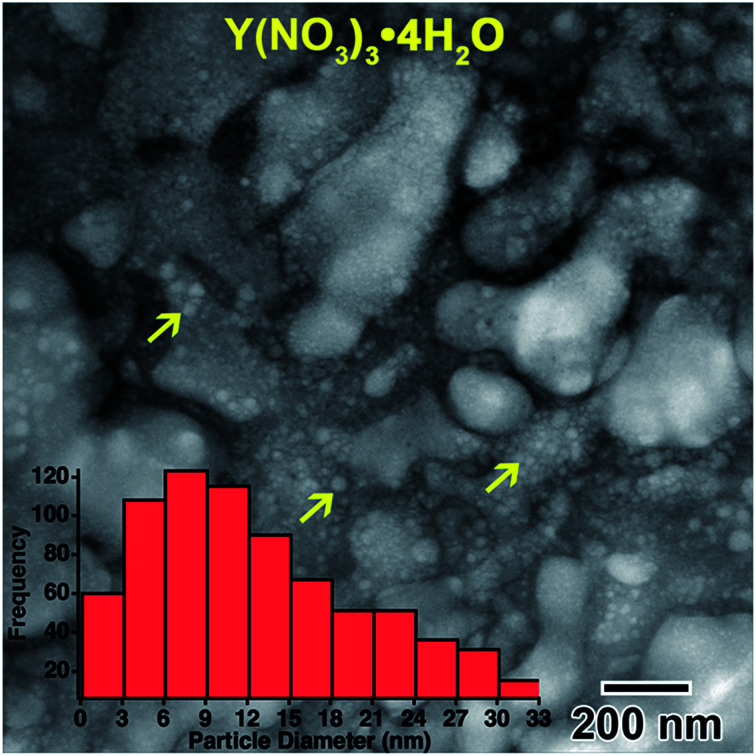
Higher magnification image showing the smaller nanoparticles, indicated by yellow arrows, located on globular features after *in situ* e^−^ irradiation of the 0.009 M Y(NO_3_)_3_·4H_2_O solution shown in Fig. 1. The inset histogram shows the size distribution of these smaller nanoparticles.

However, most nanostructures appeared unchanged. The electron diffraction pattern appeared crystalline. Crystallinity was not observed to increase after additional electron beam exposure. Reduced yttrium from the Y(NO_3_)_3_·4H_2_O solution was found generally more difficult to crystallize than metals resulting from the other salt solutions used in this study. The final electron diffraction pattern most closely matched yttrium oxide, Y_2_O_3_, which is shown in the lower left inset in Fig. 1(d). Each ring in the diffraction pattern in Fig. 1(d) was indexed, shown in Table 1. The presence of oxide indicates a vicinal combination of reduction and oxidation reactions occurring under electron beam irradiation in this solution. Based on the images, the nanostructures were initially amorphous and only crystalized during the oxidation process, without any notable restructuring to the particles themselves. Fig. 2 shows a higher magnification image taken after the *in situ* synthesis, where smaller nanoparticles are visible. The histogram in Fig. 2 shows that particles range from 1–33 nm in diameter. These smaller nanoparticles were not visible at the magnification utilized during the *in situ* synthesis, and possibly formed at around the same amount of electron beam exposure required to crystallize the material. Oxidation can be more likely when utilizing electron beam induced reduction of salt solutions as compared to chemical methods due to a higher quantity of oxidizing species present in the solution,^17^ and is therefore not unexpected. ACOM results (discussed later) indicated that the globular features are likely amorphous, while the smaller particles decorating these features in Fig. 2 are likely crystalline.

**Table 1:** Summary of the phases determined from each solution. LaCl_3_·7H_2_O (a) was 0.012 M and LaCl_3_·7H_2_O (b) was 0.005 M. Rings were numbered starting with the inner ring, which has the smallest diameter

	LaCl_3_·7H_2_O (a)	LaCl_3_·7H_2_O (b)	Y(NO_3_)_3_·4H_2_O	EuCl_3_·6H_2_O	
Indexed composition	LaCl_3_·7H_2_O (salt)	La metal	Y_2_O_3_	EuCl_3_·6H_2_O (salt)	Eu metal
Crystal system	Triclinic	Hexagonal	bcc	Monoclinic	bcc
Space group	*P*1̄	*P*6_3_/*mmc*	*Ia*3̄	*P*2/*n*	*Im*3̄*m*
Ring #1 *hkl*	1 1 0	0 1 1	2 2 2	1 0 −1	
Ring #2 *hkl*	1 2 −1	0 1 2	0 4 4		0 1 1
Ring #3 *hkl*	1 1 −2	1 1 0	2 2 6	−2 1 1	0 0 2
Ring #4 *hkl*	−1 2 1	1 1 4			1 1 2
Ring #5 *hkl*					0 2 2
Ring #6 *hkl*					0 1 3
Ring #7 *hkl*					1 2 3

#### LaCl_3_·7H_2_O

**Fig. 3:**
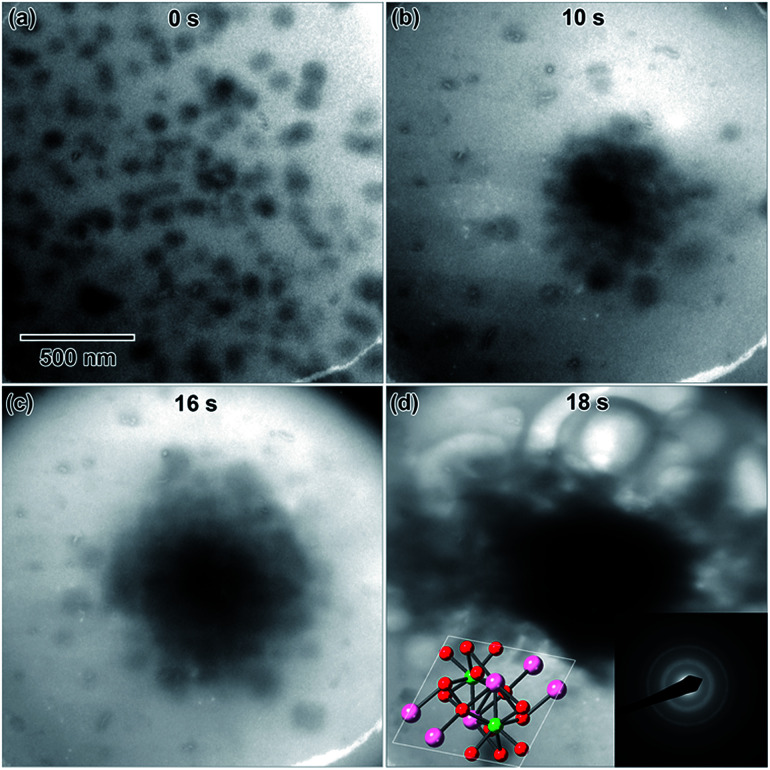
*In situ* images of nanostructure evolution in 0.012 M LaCl_3_·7H_2_O (a) solution irradiated using a converged e^−^ beam flux of 1.6 × 10^−7^ e^−^ Å^−2^ s^−1^. Frames (a)–(d) show nanostructure evolution as a function of time under a converged e^−^ beam. The final structure was determined from the SAD in (d) to be composed of small LaCl_3_·7H_2_O salt crystals. A drawing of the salt crystal structure is shown in the [001] viewing direction as an inset in (d).

**Fig. 4:**
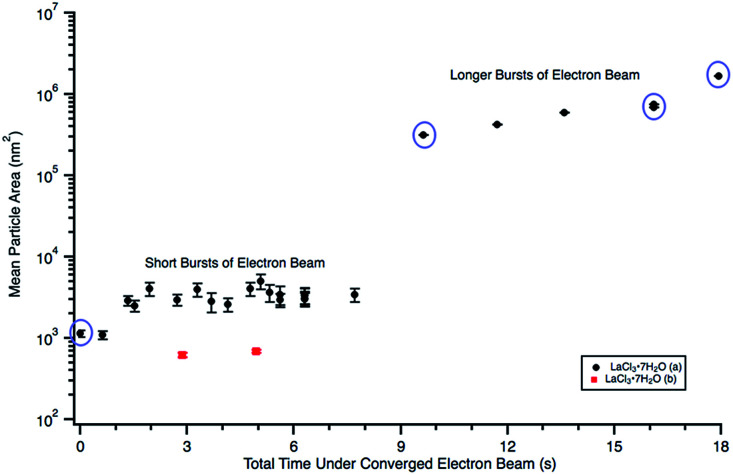
Plot showing particle size changes during *in situ* e^−^ beam irradiation in 0.012 M LaCl_3_·7H_2_O (a) (Fig. 3) and 0.005 M LaCl_3_·7H_2_O (b) (Fig. 5) solutions. Mean particle sizes corresponding to Fig. 3(a)–(d) are circled in purple. Particle sizes were only quantified using Fig. 5(b) and (c). Note that error could not be calculated for the “longer burst” data points because there was only one agglomerate in the image.

First, a 0.022 M diluted solution, prepared using a fresh (0 d old) LaCl_3_·7H_2_O salt solution, was irradiated using a converged beam flux of 3.0 × 10^−7^ e^−^ Å^−2^ s^−1^. The solution appeared very thick and already contained large, thick features that were unaltered by the electron beam. A 0.012 M solution, hereafter referred to as LaCl_3_·7H_2_O (a), was prepared from an 8 d old salt solution and irradiated using 1.6 × 10^−7^ e^−^ Å^−2^ s^−1^. The LaCl_3_·7H_2_O (a) solution initially contained a high density of spherical particles around 80 nm in diameter, Fig. 3(a). These could have formed while the TEM filament was being turned on, or they could have been present in the solution prior to any electron beam exposure. The mean particle area was quantified each time the electron beam was converged in solution, shown in Fig. 4. The data points circled in Fig. 4 correspond to the frames shown in Fig. 3. As shown in Fig. 4, short bursts, where the electron beam was only converged for typically 0.5–1.0 s, resulted in almost no particle size increase, even after 7.7 s of total converged electron beam exposure. When the pulse time was increased to 2.0–3.0 s per pulse, the particles began to agglomerate, Fig. 3(b), causing the nanostructure size to increase by two orders of magnitude. The nanoparticle agglomerate continued to increase in size with additional electron beam exposure, Fig. 3(c), eventually forming a whip-like structure in Fig. 3(d). A linear fit to the “long burst” portion of the plot in Fig. 4 gave a slope of 77 966 nm^2^ s^−1^ and an intercept of 514 307 nm^2^. A SAD pattern of the final structure is shown in the lower right inset of Fig. 3(d), which when indexed most closely matched the initial LaCl_3_·7H_2_O salt. The pattern is indexed in Table 1. This likely indicates that the particles observed in Fig. 3(a) after no electron beam exposure were nanoscale salt crystals that had crystallized out of solution. A drawing of the salt crystal structure is provided as an inset in Fig. 3(d).

A 0.012 M diluted solution prepared from a 28 d old salt solution was irradiated with 1.3 × 10^−7^ e^−^ Å^−2^ s^−1^ and formed large crystals that broke the SiN_*x*_ windows. Aging the salt solution for an additional 20 d resulted in rapid salt crystal formation under the electron beam, possibly due to evaporation of water from the original solution, effectively increasing the salt concentration.

**Fig. 5:**
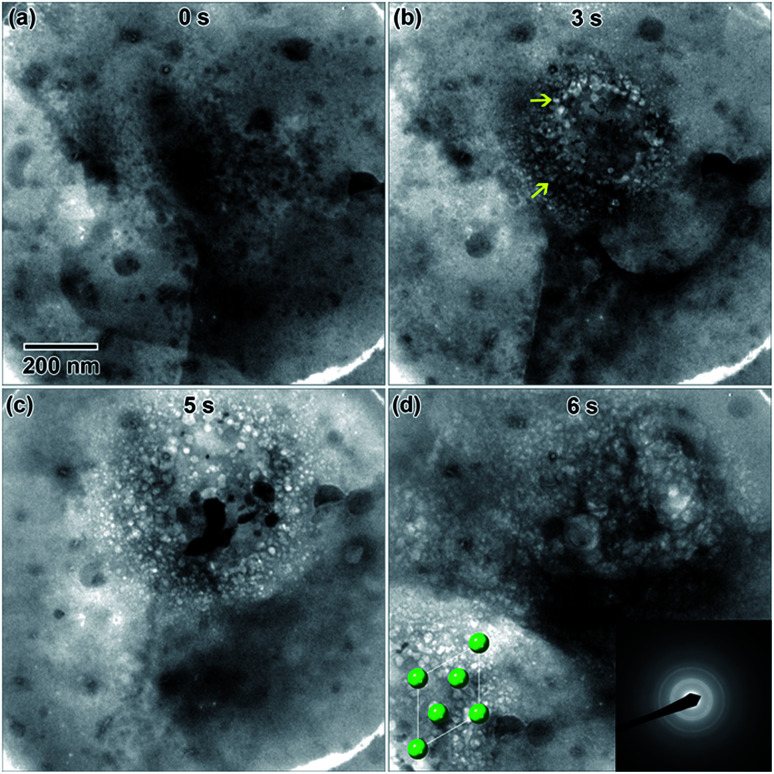
*In situ* images of nanostructure formation in 0.005 M LaCl_3_·7H_2_O (b) solution irradiated using a converged e^−^ beam flux of 1.3 × 10^−7^ e^−^ Å^−2^ s^−1^. Frames (a)–(d) show nanostructure evolution as a function of time under a converged e^−^ beam. Arrows in (b) indicate initial nanoparticle formation. The final structure was determined from the SAD in (d) to be lanthanum metal. A drawing of the lanthanum metal crystal structure is shown in the [001] viewing direction as an inset in (d).

A 0.006 M solution was prepared from a 28 d old salt solution, but still appeared too thick to observe nanostructure formation. Crystalline nanostructure formation was observed in a 0.005 M solution, hereafter referred to as LaCl_3_·7H_2_O (b), which was prepared from a 28 d old salt solution and irradiated with the same 1.3 × 10^−7^ e^−^ Å^−2^ s^−1^ flux. LaCl_3_·7H_2_O (b) also contained an initial distribution of particles or slightly viscous liquid, shown in Fig. 5(a). These were possibly due to electron beam exposure before imaging when the TEM filament was being turned on, or were conceivably present in the initial 28 d old solution. After converging the electron beam on the solution for a total 3 s, particles with a mean area of 621 ± 38 nm^2^ (14 nm diameter) began to appear in the solution. Most of the particles appear under the location where the electron beam was converged, marked with arrows in Fig. 5(b). No particle growth was observed with additional electron exposure; after 5 s, the mean particle area was 688 ± 30 nm^2^ (14.8 nm in diameter). Alteration of the overall nanostructure appeared to occur after a total of 6 s of exposure, Fig. 5(d). Electron diffraction showed that the final structure was crystalline. The diffraction pattern indexed best with lanthanum metal, which is drawn as an inset in Fig. 5(d). The pattern is indexed in Table 1. Even though there could have been an initial distribution of salt particles in the solution (see Fig. 5(a)), all the salt ions have been converted to lanthanum metal under electron beam irradiation, the result predicted by the reaction in eqn (2).

This is contrary to LaCl_3_·7H_2_O (a), where the structure of initial salt particles was extremely stable under the electron beam. LaCl_3_·7H_2_O (a) contained an initial distribution of much larger particles and a less viscous fluid than the LaCl_3_·7H_2_O (b) solution, which may have resulted in higher stability of the salt phase under electron beam irradiation. While the redox potential is the same for reduction of La^3+^ than Y^3+^, the LaCl_3_·7H_2_O solution was found to form crystalline nanostructures much more readily compared to the Y(NO_3_)_3_·4H_2_O solution, which required the highest electron dose of all three salt solutions to crystallize.

#### EuCl_3_·6H_2_O

First, a 0.013 M solution as prepared from a 15 d old EuCl_3_·6H_2_O salt solution. Large crystals several hundred microns in size formed immediately upon electron beam exposure and burst the SiN_*x*_ windows. A 0.009 M solution was prepared from a 15 d old salt solution and also caused large crystals to burst the SiN_*x*_ windows upon electron beam exposure. A 0.005 M solution was prepared from the same 15 d old salt solution and irradiated under the electron beam with a converged beam flux of 2.5 × 10^−7^ e^−^ Å^−2^ s^−1^. Nanostructures were nearly identical in appearance to the 0.005 M LaCl_3_·7H_2_O solution, Fig. 5, which crystalized in a metal phase. The 0.005 M EuCl_3_·6H_2_O solution contained crystalline nanostructures, but the electron diffraction pattern could not be indexed with database structures, possibly due to the presence of preferred orientation and multiple phases.

**Fig. 6:**
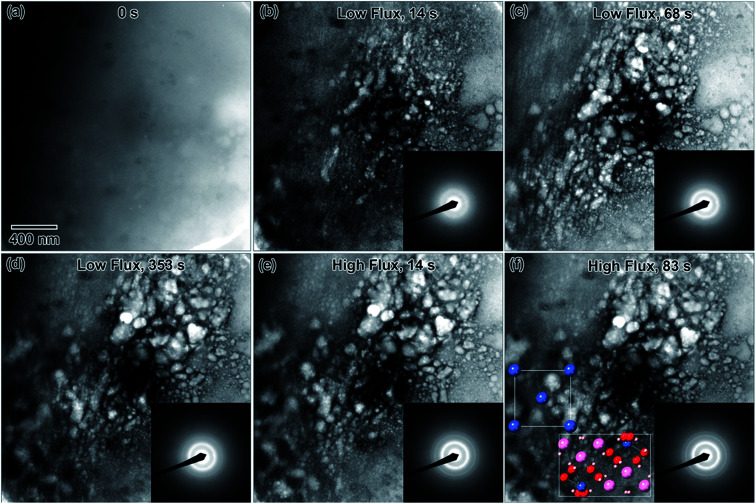
*In situ* images of nanostructure formation in 0.002 M EuCl_3_·6H_2_O solution irradiated with two different converged e^−^ beam fluxes. The converged electron beam flux was 8.2 × 10^−8^ e^−^ Å^−2^ s^−1^ for frames (b)–(d) and increased to 2.4 × 10^−7^ e^−^ Å^−2^ s^−1^ for frames (e) and (f). Frames (a)–(f) show nanostructure formation and crystallization from solution as a function of time under the converged e^−^ beam. SAD patterns were taken *in situ* after the listed total time under the converged e^−^ beam. The final structure was determined from the SAD in (f) to be Eu metal and likely also some of the initial salt, EuCl_3_·6H_2_O. Drawings of the Eu (blue) and salt crystal (multicolor) structures are shown in the [001] viewing direction as an inset in (f).

**Fig. 7:**
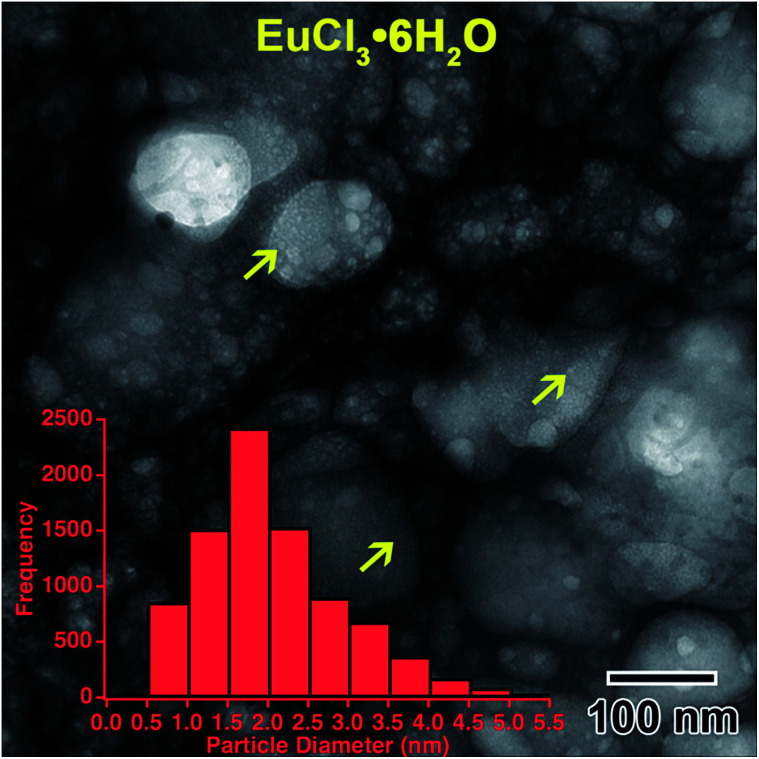
Higher magnification image showing the smaller nanoparticles, indicated by yellow arrows, located on globular features after *in situ* e^−^ irradiation of the 0.002 M EuCl_3_·6H_2_O solution shown in Fig. 6. The inset histogram shows the size distribution of these smaller nanoparticles.

Rapid crystallization of the solution was eventually avoided by preparing a 50% dilute EuCl_3_·6H_2_O salt solution, with a molarity of 0.069 M. A separate 0.002 M diluted solution was prepared from 3 d old 50% dilute salt solution. The salt solution was heated to 50 °C to ensure the salt mixture was fully dissolved before preparing the diluted solution. Only the diluted solution appeared in the microfluidic cell prior to converging the electron beam, Fig. 6(a). Initially, the electron beam was converged with a beam flux of 8.2 × 10^−8^ e^−^ Å^−2^ s^−1^. The structure in Fig. 6(b) formed after converging the electron beam for 14 s. Fig. 6(b)–(d) shows the progression of the nanostructure and its diffraction pattern for this flux, from 14 to 353 s. The diffraction pattern in Fig. 6(b) indicates that only an amorphous structure formed after 14 s. However, the pattern in Fig. 6(c) indicates that some crystallization has occurred after 68 s. The nanostructures appear to agglomerate into larger structures between 68 and 353 s of electron exposure, Fig. 6(c) and (d). Interestingly, the rings do not appear anymore defined in the diffraction pattern taken after 353 s, suggesting no change in the crystallinity. To increase the crystallinity, the converged electron beam flux was increased to 2.4 × 10^−7^ e^−^ Å^−2^ s^−1^. The rings in the diffraction pattern appear more defined after 14 s of the electron beam converged on the solution with this flux, Fig. 6(e), though the nanostructures in Fig. 6(e) appear unchanged from Fig. 6(d). This more defined diffraction pattern indicates an increase in crystallinity. After 83 s of converged electron beam exposure at the higher flux, Fig. 6(f), the nanostructures again appear unchanged, but the electron diffraction pattern seemed slightly more defined. The final diffraction pattern in Fig. 6(f) was indexed and determined to most closely match europium metal, except for the thick inner ring, which is presumed to be EuCl_3_·6H_2_O salt. The highest intensity salt rings, 101̄ and 2̄11, have *d*-spacings of 6.21 and 3.41 Å, respectively, and the inner-most europium metal ring, 011, has a *d*-spacing of 3.24 Å. This means that the highest intensity diffraction rings in europium salt solution would have a smaller diameter than the smallest diameter ring in europium metal, which fits this prediction. The formation of Eu metal was predicted by the reaction in eqn (2). The presence of salt post irradiation is presumed to be a result of larger salt crystals in solution that were unable to be reduced to metallic structures by the electron beam. Final solution salt crystals were observed for all salt solutions that were not very dilute. A similar result was observed in the La solution above and suggests high stability larger salt crystals. All diffraction indexing is provided in Table 1. The inset in Fig. 6(f) shows drawings of the cubic metal and salt crystal structures. Fig. 7 shows a higher magnification image taken after the *in situ* synthesis, where smaller nanoparticles are visible. The histogram in Fig. 7 shows that particles range from 0.5–5.0 nm in diameter, smaller than the nanoparticles observed in the Y solution (Fig. 2). These smaller nanoparticles were not visible at the magnification utilized during the *in situ* synthesis. The globular features in Fig. 6 appear similar to the globular features observed in the Y solution in Fig. 1. If we extend the ACOM results on the Y solution nanostructures to this case, it is possible that the smaller particles in Fig. 7 are the crystalline components. Crystalline nanostructures readily formed in the EuCl_3_·6H_2_O solution, which was expected to form nanostructures easier than the other two salt solutions due to the lower redox potential of Eu^3+^.

#### ACOM analysis

**Fig. 8:**
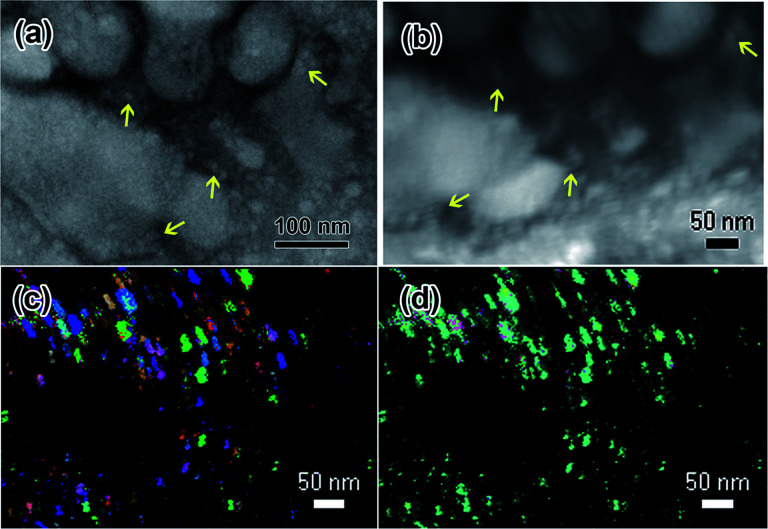
PED scans of nanostructures that formed under electron beam irradiation of the 0.009 M Y(NO_3_)_3_·4H_2_O solution from Fig. 1. (a) shows a BF TEM image of the region where the ACOM scan was done, (b) shows the VBF image produced by the ACOM software, which includes slight drift that occurred during the scan, (c) shows an overlay of *y*-orientation and index, and (d) shows an overlay of *z*-orientation and index. The *z*-direction is parallel to the electron beam. ACOM data were indexed using a bcc Y_2_O_3_ crystal structure, as indexed in the electron diffraction pattern shown in Fig. 1(d). Colors shown in (c) and (d) correspond to cubic crystallographic directions as follows: green = 100, red = 001, blue = 111. Arrows in (a) and (b) indicate the corresponding locations of several particles in the BF and VBF images.

In all three solutions, the crystalline features were indistinguishable from the amorphous structures using electron diffraction alone due to many of the synthesized particles being <50 nm in diameter in many cases, and frequent vicinal phases. ACOM maps were utilized to separate nanoparticles with different phases in the final structures. By way of experimental example, results are shown here for the nanostructures formed in the Y(NO_3_)_3_·4H_2_O solution in Fig. 8. In this case, the data were indexed using Y_2_O_3_, which is the structure that most closely matched the diffraction pattern in Fig. 1(d). Fig. 8(a) shows both the Bright Field (BF) TEM image of the scan area. The same area is shown in Fig. 2. Fig. 8(b) shows the Virtual Bright Field (VBF) TEM image created from the ACOM data. Because the scans were collected over several hours, drift resulted and can be seen in this solution in Fig. 8(b). The nanostructure appeared to be entirely solid after the final electron beam exposure, so the drift is likely due to the stage itself, and not remaining liquid.

Several nanoparticles present in the BF TEM image are indicated with arrows in the VBF image created by the ACOM data, see Fig. 8(c) and (d). The *y*-orientation is parallel to the SiN_*x*_ windows and the *z*-orientation is parallel to the electron beam. Black regions in both figures indicate a purely amorphous or absent diffraction pattern. The black regions appear to align with the large globular features that were observed in this solution, indicating that these structures could be amorphous contributions to the electron diffraction pattern in Fig. 1(d). The brightly colored crystalline regions in Fig. 8(c) and (d) appear to correlate with the smaller particle locations indicated by arrows in the TEM image in Fig. 8(a) and the VBF image in Fig. 8(b). The *y*-orientation of the smaller particles appears to vary, while the *z*-orientation appears to be mostly the same. This suggests that the particles may have grown from one of the amorphous SiN_*x*_ windows of the microfluidic cell in an epitaxial fashion.

Overall, the studies with ACOM were successfully utilized to deconvolute amorphous and crystalline nanoparticle components which formed under the electron beam in the Y(NO_3_)_3_·4H_2_O solution. *In situ* electron diffraction patterns provided crystallographic phase information for all phases present in the entire nanostructure synthesized under the electron beam. Post irradiation, ACOM analysis collected electron diffraction patterns at specific areas within the nanostructure, which were then indexed using the known phases present in the structure and used to glean information about growth orientation and phase identification of specific nanoparticles within the larger structure.

However, experimental difficulties still remain in utilizing ACOM with the liquid cell. Primarily, the aligning of the eucentric height correctly in the microscope. In many cases, nanostructures will grow epitaxially perpendicular to the plane of one or both cell windows, making the *z*-height alignment difficult. The smaller particles are likely present throughout the microfluidic cell, presenting the same issue. In all attempts at ACOM in the microfluidic cell, the resolution in the VBF image was much lower than in the BF image, indicative of poor *z*-height alignment. Poor *z*-height alignment could result in incorrect assessment of the size and reliability of the crystalline features.

In the supplemental file, an ACOM scan performed on LaCl_3_·7H_2_O (b) is shown. ACOM was attempted on the EuCl_3_·6H_2_O solution as well, but the resolution was very poor and the data could not be indexed. In addition to *z*-height alignment issues, the amorphous features and/or liquid remaining in the microfluidic cell present an amorphous background in each of the recorded diffraction patterns in the dataset. This results in systematically lower reliability values than desirable for all the data collected. In many cases, crystalline particles are also smaller than the probe size (between 5–10 nm), which could result in multiple orientations being recorded in one step if two particles are present in the same spot diameter.

Despite these challenges, ACOM provided useful insight into the crystallinity and epitaxial growth of various nanostructures appearing in the Y(NO_3_)_3_·4H_2_O salt solution.

### Flow cell experiments

#### Y(NO_3_)_3_·4H_2_O

A 0.1009 M solution was initially flowed into the stage at 100 μL h^−1^ for 30 min without converging the electron beam. When no nanostructures were observed, the flow rate was increased to 300 μL h^−1^ for the remainder of the experiment to see if more unreacted solution flowing through the cell would increase the size of precipitates to the point where they would be visible inside the TEM. Even after increasing the flow rate, nanostructure formation was only observed after converging the electron beam inside the solution, as was observed in the close cell experiments.

**Fig. 9:**
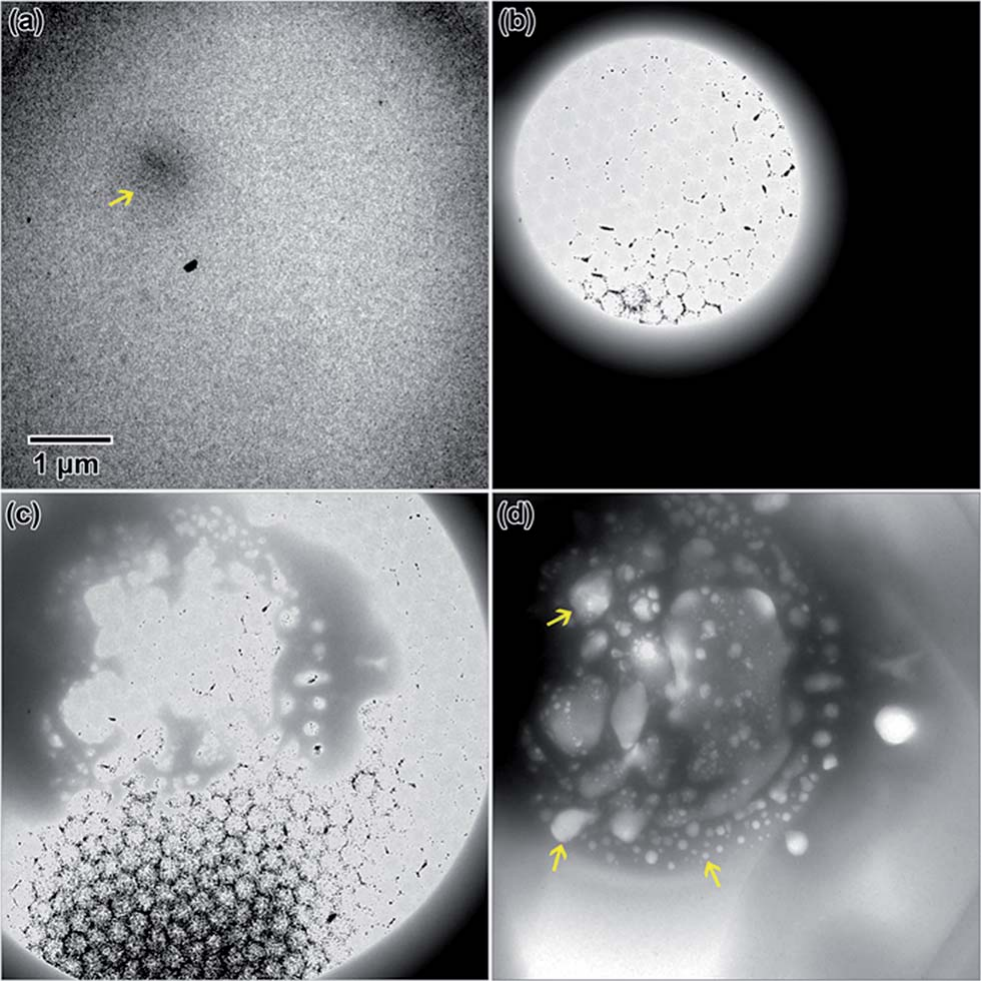
*In situ* nanostructure evolution in the flow cell from Y(NO_3_)_3_·4H_2_0 solution: (a) initial nanostructure nucleated by focusing the electron beam, indicated by arrow, (b) the electron beam converged to a point on the nanoparticle in solution, (c) large microstructure formation, and (d) the final microstructure resulting from the focused electron beam, with examples of possible cavities indicated by arrows.

An example of electron beam convergence and the subsequent reaction is shown in Fig. 9(b). The initial nanostructure, indicated by an arrow in Fig. 9(a), formed after only a few seconds of electron exposure and was 750 nm in diameter. That structure eventually grew into a spherical nanostructure 4.4 μm in diameter, shown in Fig. 9(b) and (c). The honeycomb structure observed in Fig. 9(b) and (c) is an artifact due to saturation of the CCD camera. The final nanostructure, in Fig. 9(d) appeared to be a complex structure containing internal nanoscale features, possibly cavities, with dimensions ranging from 25 nm–1 μm in length.

#### LaCl_3_·7H_2_O

**Fig. 10:**
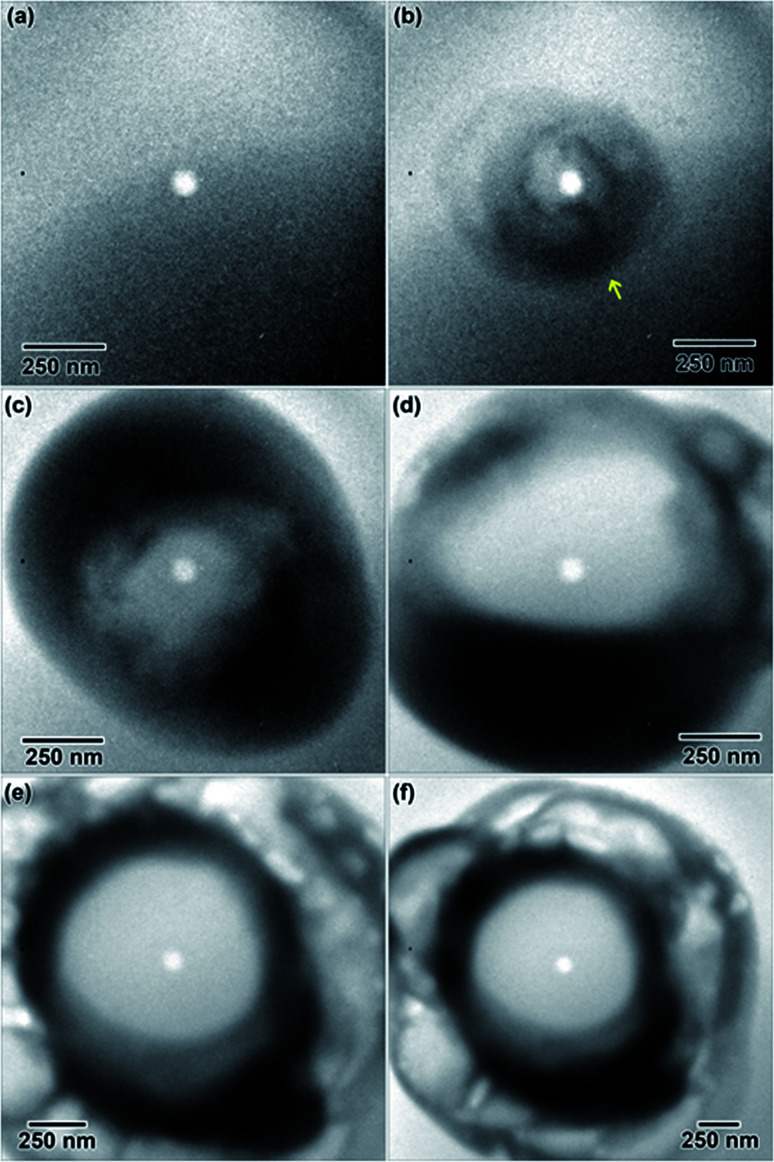
*In situ* nanostructure evolution in the flow cell from LaCl_3_·7H_2_O solution. (a) shows the initial cell containing unreacted liquid. (b)–(e) show the microstructure after the electron beam was repeatedly converged on the location where initial nanostructure formation was observed, indicated by an arrow in (b), to form the final structure shown in (e).

A 0.0479 M solution and deionized water were flowed into the stage at 100 μL h^−1^ each. In this solution, electron irradiation initially resulted in nanoparticles like the one indicated by an arrow in Fig. 10(b) after about 10 s of electron exposure. After about 20 s of electron exposure, the structure in Fig. 10(d) was observed, eventually evolving to the 2.3 μm in diameter final microstructure, shown in Fig. 10(f), after around 1 min of electron exposure. The structure appeared to form in a columnar fashion, extending between both SiN_*x*_ windows of the microfluidic cell. Similar structures were reproduced in the same solution by focusing the electron beam on different areas of the microfluidic chamber.

#### EuCl_3_·6H_2_O

**Fig. 11:**
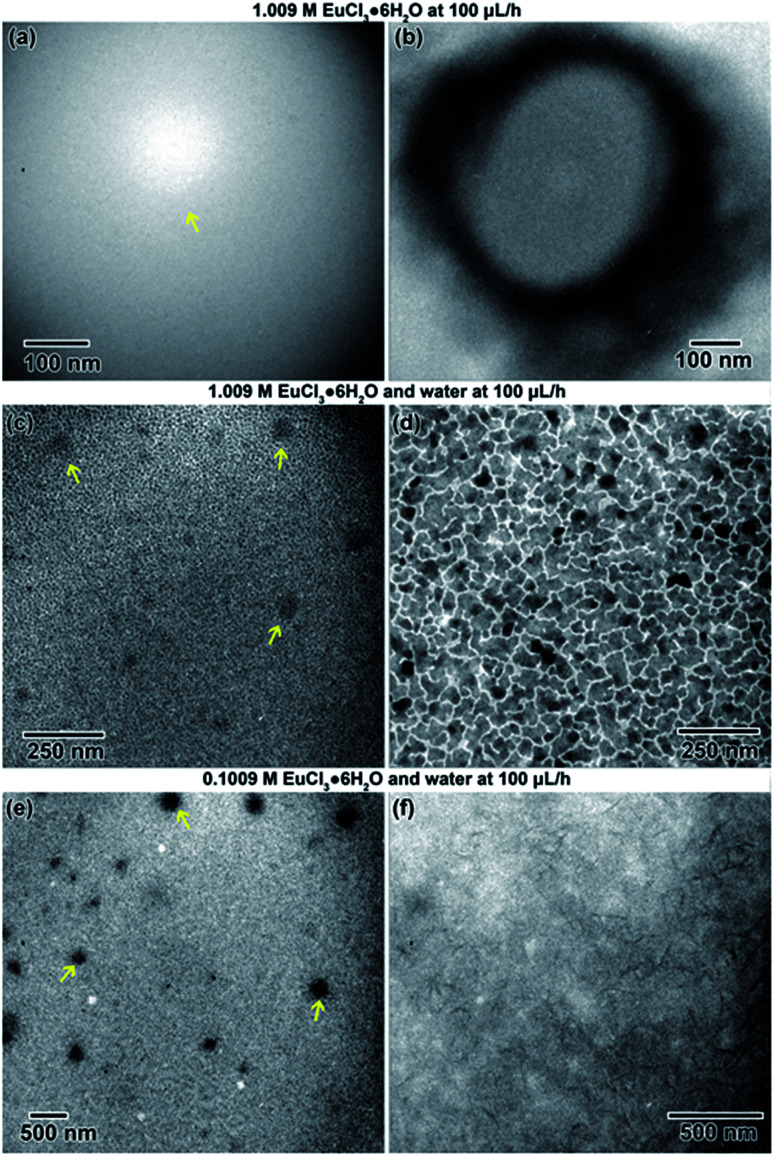
*In situ* nanostructure evolution in the flow cell from EuCl_3_·6H_2_0 solution at the various concentrations and flow conditions explored in this study before (left) and after (right) converging the electron beam to form the final structure. Example precipitates observed in the liquid solution are indicated by arrows.

Two concentrations and two flow conditions were utilized to study the Eu-based salt solution. In the first experiment, a 1.009 M solution was flowed into the stage at 100 μL h^−1^. The electron beam was focused on the sample resulting in a what appeared to be a solid columnar structure precipitating from the solution. The precipitate started in the region indicated by an arrow in Fig. 11(a), eventually reaching a stable structure 414 nm in diameter Fig. 11(b).

In the second experiment, the same solution (1.009 M), at the same flow rate (100 μL h^−1^) was used, but with the addition of deionized water flowed (100 μL h^−1^) into the second inlet line. This diluted the sample solution in the microfluidic chamber by approximately half. Some initial nanostructures around 45 nm in diameter, shown with arrows in Fig. 11(a), appeared in the solution after focusing the beam After about 1 min of exposure to the electron beam, the solution began to electroplate onto the SiN_*x*_ windows of the microfluidic cell. The electroplated regions, shown in Fig. 11(b), were an average of 60 nm in size, but were interconnected.

In the third experiment, a 0.1009 M solution (10× less) and water were both flowed into the microfluidic cell simultaneously at 100 μL h^−1^. Initially converging the beam for only a few seconds resulted precipitate structure formation in some cases. In one case, nanostructures formed with an average diameter of approximately 169 nm, indicated by arrows in Fig. 11(e). After additional electron exposure, dendritic structures, which appeared to be stuck to the SiN_*x*_ windows in a stable form, formed with an average length of around 116 nm. These are shown in Fig. 11(f).

### Comparison of closed and flow experiments

Interestingly, the three different rare earth solutions behaved differently under closed and flow cell conditions. In flow, the Y solution (Fig. 9) formed a large structure covered in smaller nanoparticles, similar to the globular features littered with smaller nanoparticles in the closed cell (Fig. 2). The La and Eu flow experiments did not show similarities with microstructures observed in close cell results. In the flow cell experiments, the salt solutions were diluted *in situ* and received electron beam exposure while being diluted. This will result in some nanostructure formation during the dilution process.

The liquid cell experimental parameters dictate much of the final crystalline product formation. The closed cell experiments allow for easy determination of crystallinity because the nanostructures do not move around the liquid cell during imaging, but the flow cell experiments probably provide a move realistic idea of how nanostructures might form in bulk electron beam radiolysis. In the static condition, the solution often because viscous after electron beam exposure, preventing any movement. The wide variety of structures observed in both closed and flow experiments indicates the complexity of rare earth nanostructure synthesis. The salts were shown to form metallic and oxide structures, in addition to maintaining their original salt crystal structure in some cases. Individual crystalline particles were only observed scattered among larger amorphous features. This is in contrast to *in situ* work on synthesis of Ag^15^ and PbS^14^ nanoparticles, for example, where individual, spherical nanoparticles were observed nucleating and diffusing around the solution.

Nucleation parameters were found particularly difficult to control in the rare earth solutions, as there seemed to be a threshold electron beam flux and pulse time at which large, complex structures would rapidly appear. Nucleation and growth of individual particles into larger structures was never observed, but crystallinity was observed to increase in each salt solution with additional electron dose, even with little modification to the nanostructure. Agglomerates similar to those observed in the La solution, Fig. 3, were also observed after gamma-irradiation induced synthesis of UO_2_ nanoparticles.^11^ Electron-beam induced radiolysis of aqueous Pd salt^17^ also resulted in nucleation of individual particles, followed by growth into a flower-like structure, forming larger, complex nanostructures similar to some seen in this rare earth *in situ* microfluidic study.

## Conclusions

In summary, electron beam irradiation was successfully utilized for reduction and crystallization of three rare earth-based salt solutions in real-time, while stagnantly contained or flowing through an *in situ* liquid cell TEM. The solutions studied were yttrium(iii) nitrate hydrate (Y(NO_3_)_3_·4H_2_O), europium(iii) chloride hydrate (EuCl_3_·6H_2_O), and lanthanum(iii) chloride hydrate (LaCl_3_·7H_2_O). Metal nanostructures formed in the EuCl_3_·6H_2_O and LaCl_3_·7H_2_O solutions, as predicted by the reduction reaction in eqn (2). The Y(NO_3_)_3_·4H_2_O solution was difficult to crystallize and formed both precipitated salt and an oxide structure in addition to the reduced metal, indicating that both reduction and oxidation reactions occurred during electron beam irradiation within nanometers of each other.

ACOM was successfully utilized to deconvolute the phase, orientation, and location of these complex nanoparticle components, while in a microfluidic TEM cell. *In situ* microfluidic methods for creating rare earth-based nanostructures is complex compared to previous noble metal studies and highly dependent on the initial concentration of the salt solution, age of the solution, electron beam flux, and electron beam pulse time. The amorphous fraction was found to be directly dependent on total electron beam dose in all cases, but some salts crystallized more readily than others. Resulting microstructures seemed highly dependent on flow rate when the microfluidic cell was operated in this condition.

Ongoing work is focused on tuning the variables of charged particle beam mass, energy, and intensity, as well as microfluidic flow rate, solution concentrations, surface chemistry, and chamber dimensions. These studies all contain the goal of understanding the governing mechanisms of nanostructure formation and subsequently predicting formation conditions for mining, processing, and recycling applications of these rare earth compounds.

## Conflicts of interest

There are no conflicts to declare.

## Supplementary Material

NA-001-C9NA00197B-s001
