# Supplementary material for: Synthesis of complex rare earth nanostructures using in situ liquid cell transmission electron microscopy
Source: Nanoscale Adv. 2019 Apr 18;1(6):2229–39. doi: 10.1039/c9na00197b (PMC9418461; doi:10.1039/c9na00197b)
Supplement: NA-001-C9NA00197B-s001 [file NA-001-C9NA00197B-s001.pdf]

Electronic Supplementary Material (ESI) for Nanoscale Advances.
This journal is © The Royal Society of Chemistry 2019

Final Electron Diffraction Patterns

$LaCl_3 \cdot 7H_2O$ (a)

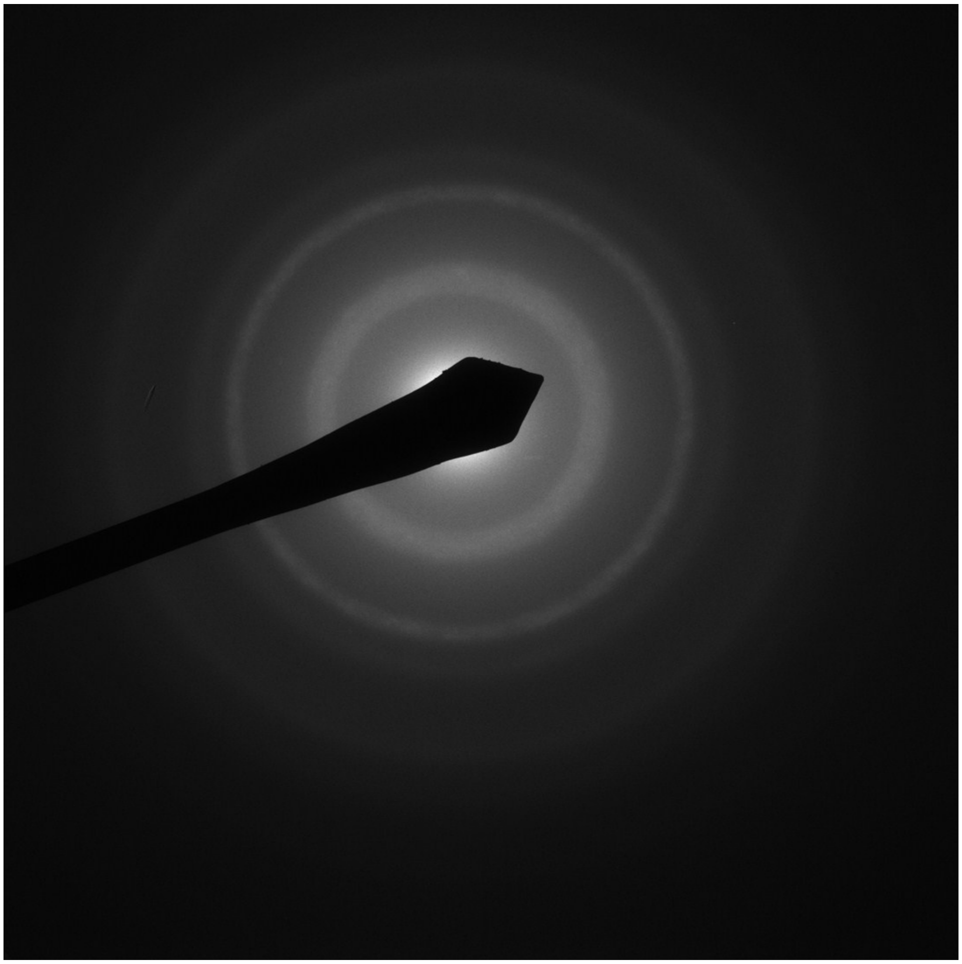

$LaCl_3$•$7H_2O$ (b)

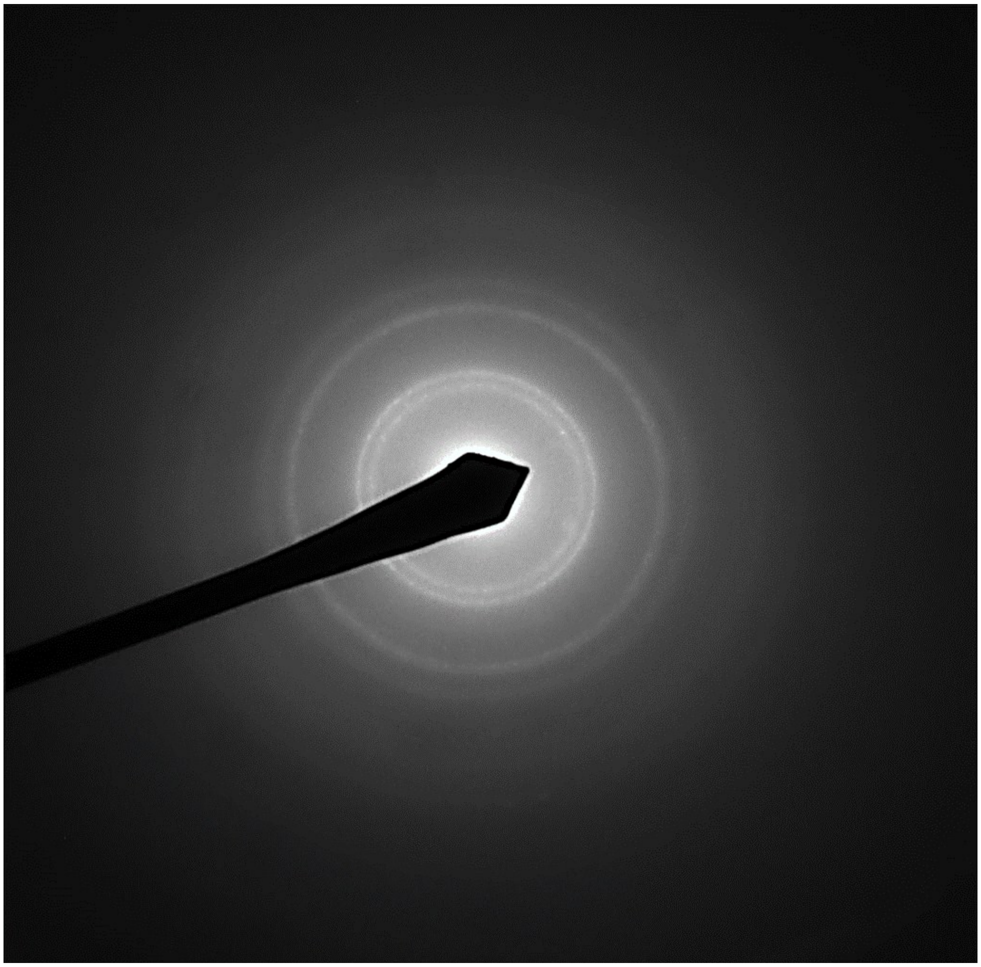

$0.009\ M\ Y(NO_3)_3 \bullet 4H_2O$

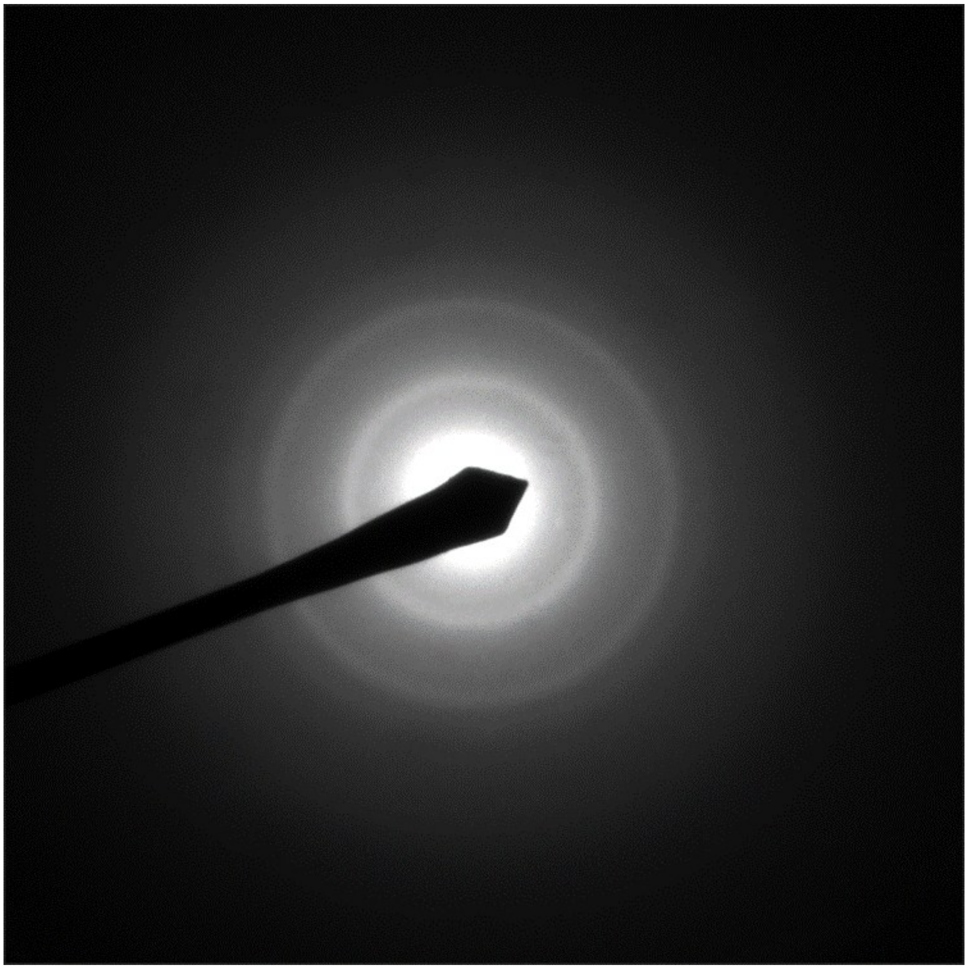

0.002 M $EuCl_3 \bullet 6H_2O$

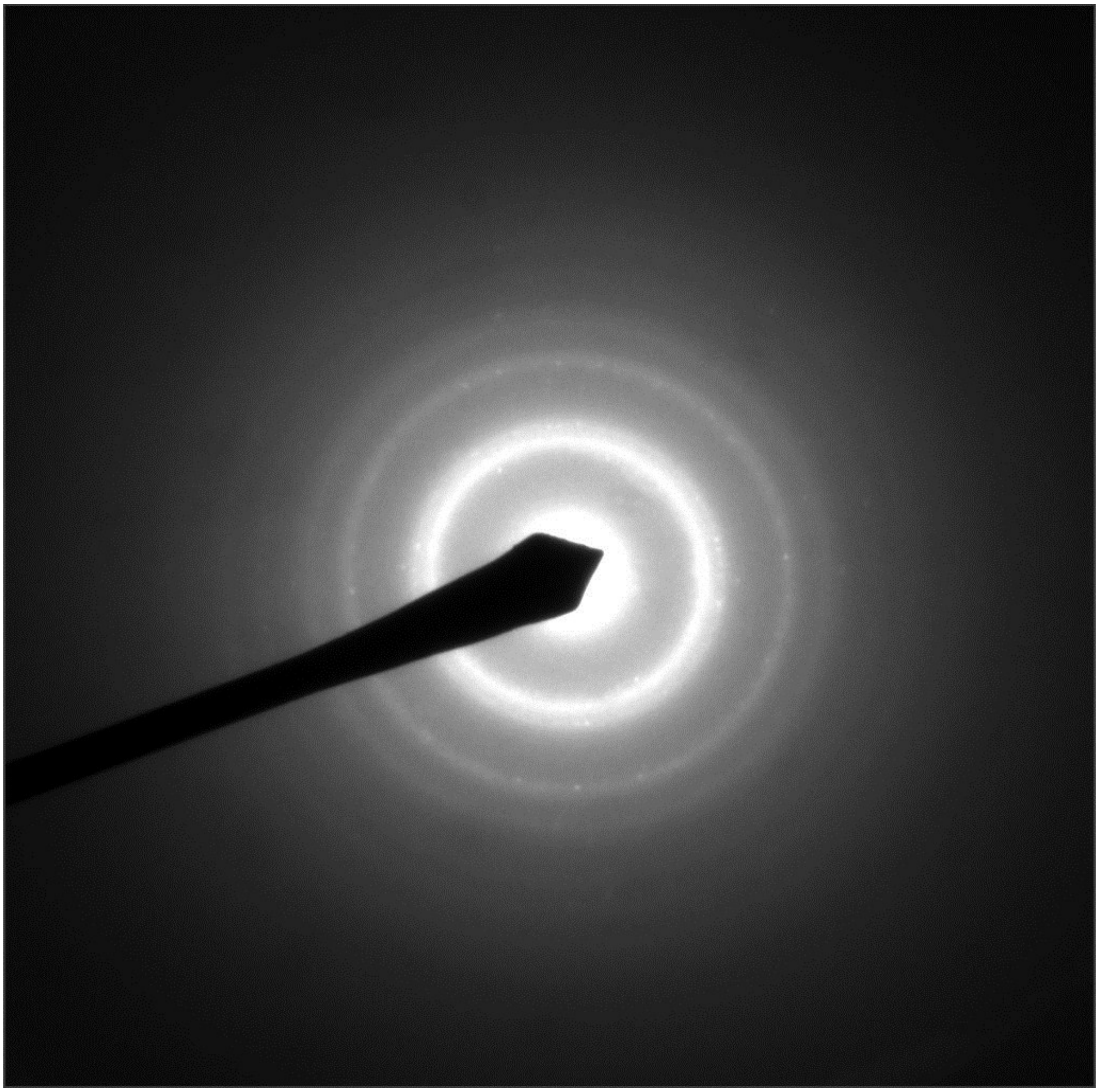

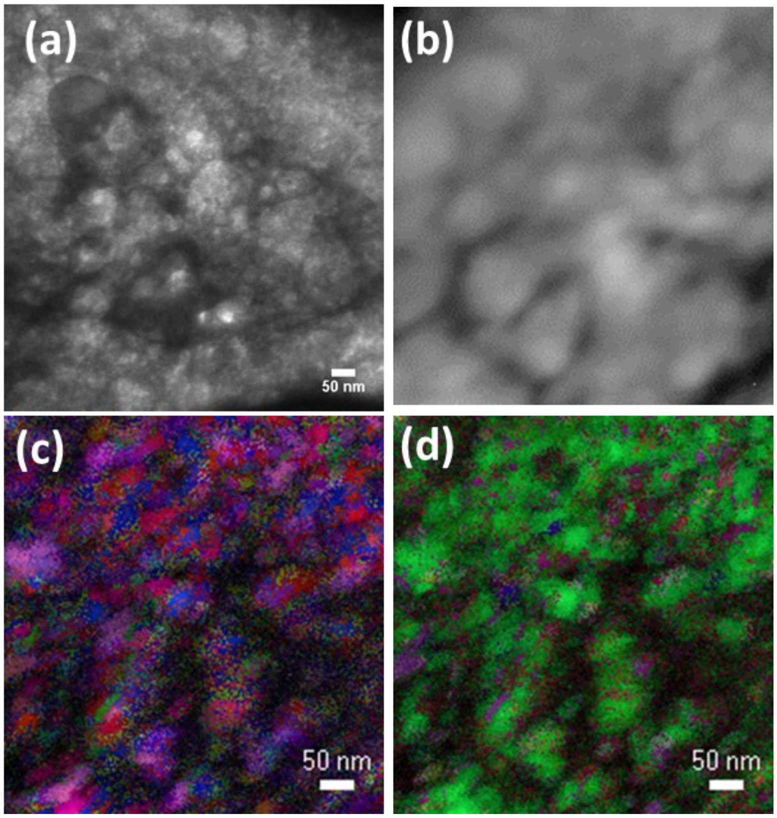

PED scans of nanostructures that formed under electron beam irradiation of the $LaCl_3$•$7H_2O$ (b) solution. (a) shows a bright field TEM image of the region where the PED scan was done, (b) shows the virtual bright field image produced by the PED software, which includes slight drift that occurred during the scan, (c) shows an overlay of y-orientation and index, and (d) shows an overlay of z-orientation and index. The z-direction is parallel to the electron beam. PED data were indexed using a hexagonal La metal crystal structure, as indexed in the electron diffraction pattern shown in Fig 2(d). Colors shown in (c) and (d) correspond to hexagonal crystallographic directions as follows: green = $2\bar{1}\bar{1}0$, red = 0003, blue = $30\bar{3}0$.

Ring numbering starts with the smallest radius ring (inside ring). In the radius ratio calculations, r_x refers to the ring # on the current row in the table. For example, in the "Ring #3", "r_2/r_x" cell of the Eu table, the radius of Ring #2 (r_2) was divided by the radius of Ring #3 (r_3) to get 0.69. The %diff values are the percent difference between the measured r_2/r_x and literature r_2/r_x.

	$EuCl_3$•$6H_2O$							
	r_2/r_x	% diff	r_3/r_x	% diff	r_4/r_x	% diff	phase 1 *hkl*	phase 2 *hkl*
**Ring #1**							1 0 -1	
**Ring #2**	1.00							0 1 1
**Ring #3**	0.69	2%	1.00				-2 1 1	0 0 2
**Ring #4**	0.58	1%	0.84	3%	1.00			1 1 2
**Ring #5**	0.49	3%	0.70	1%	0.83	4%		0 2 2
**Ring #6**	0.44	1%	0.64	1%	0.76	2%		0 1 3
**Indexed Composition**							$EuCl_3$•$6H_2O$ (salt)	Eu metal
**Crystal System**							monoclinic	bcc
**Space Group**							*P 2/n*	*I m -3 m*

*In this case, the first "thick" ring is assumed to be salt, of which the first, highest intensity ring, should have a larger d-spacing than any planes in Eu-metal and would thus have a smaller radius.

	$LaCl_3$•$7H_2O$ (a)						
	r_1/r_x	% diff	r_2/r_x	% diff	r_3/r_x	% diff	phase 1 *hkl*
**Ring #1**	1						1 1 0
**Ring #2**	0.62	3%	1				1 2 -1
**Ring #3**	0.53	6%	0.85	3%	1		1 1 -2
**Ring #4**	0.40	6%	0.64	3%	0.76	1%	-1 2 1
**Ring #5**	0.35	6%	0.56	3%	0.66	1%	3 0 -1
**Indexed Composition**				$LaCl_3$•$7H_2O$ (salt)			
**Crystal System**				triclinic			
**Space Group**				*P-1*			

*The 012 ring is missing from the salt structure, possibly preferred orientation

	$LaCl_3$•$7H_2O$ (b)						
	**r_1/r_x**	**% diff**	**r_2/r_x**	**% diff**	**r_3/r_x**	**% diff**	**phase 1 *hkl***
**Ring #1**	1						0 1 1
**Ring #2**	0.86	5%	1				0 1 2
**Ring #3**	0.56	6%	0.65	1%	1		1 1 0
**Ring #4**	0.48	6%	0.55	1%	0.84	1%	1 1 4
**Indexed Composition**	La metal						
**Crystal System**	hexagonal						
**Space Group**	$P6_3/m\ m\ c$						

	$Y(NO_3)_3$•$4H_2O$				
	**r_1/r_x**	**% diff**	**r_2/r_x**	**% diff**	**phase 1 *hkl***
**Ring #1**	1				2 2 2
**Ring #2**	0.60	2%	1		0 4 4
**Ring #3**	0.51	2%	0.85	1%	2 2 6
**Indexed Composition**	Y_2O_3				
**Crystal System**	bcc				
**Space Group**	*I a -3*				
